# Influence of anterior open bite on oral health-related quality of life. A systematic review

**DOI:** 10.4317/jced.62398

**Published:** 2025-01-01

**Authors:** Jazmín Estephany Rodriguez-Huaringa, Gloria Ximena Jassmin Vargas-Mori, Luis Ernesto Arriola-Guillén

**Affiliations:** 1 School of Dentistry, Universidad Científica del Sur, Lima, Perú; 2 Division of Orthodontics, School of Dentistry, Universidad Científica del Sur, Lima, Perú

## Abstract

**Background:**

Anterior open bite is a malocclusion caused by genetic and environmental causes that affect esthetics, occlusion, and speech, impacting the quality of life in children, adolescents, and adults. However, to date, no systematic review has analyzed the influence of anterior open bite (AOB) on oral health-related quality of life.

**Material and Methods:**

This systematic review conducted a comprehensive search in the MEDLINE (via PubMed), EBSCO, SCOPUS, and LILACS databases until September 30, 2023. A total of 321 articles were identified across these databases. After applying the selection criteria, 13 articles were selected for full-text evaluation. Ultimately, only six studies, all cross-sectional, were included in the review. The Newcastle-Ottawa Scale was utilized to assess the risk of bias in these studies.

**Results:**

Six studies have found that AOB affects quality of life. In adults, it has a greater impact on women, especially on psychological and social well-being. In children, negative effects are observed in symptoms, function, and psychological aspects, with poor outcomes. One study found no significant differences in quality of life in children with AOB.

**Conclusions:**

The review indicates that AOB (anterior open bite) significantly impacts quality of life, particularly in psychological and functional areas such as speech, eating, and smiling. This information is essential for both orthodontists and patients. While most studies show moderate bias, further well-designed research is recommended.

** Key words:**Open bite, oral health-related quality of life, review.

## Introduction

Anterior open bite (AOB) is a malocclusion of genetic or environmental etiology aggravated by unhealthy habits, it is characterized by the lack of contact between the edges of the upper and lower incisors (1,4). There are two types of open bites, dentoalveolar and skeletal, being the second one that needs a more complex treatment due to its severity (5), besides this last one has morphogenetic factors that involve to difficulties in the stomatognathic development and for having a more complex treatment that is usually surgical, except when camouflage is given as a treatment alternative, but they have a high potential for relapse, which is why some patients choose not to be treated but in some cases may affect their social relations and probably their quality of life (6,7).

Individuals with AOB present vertical predominant growth and usually have convex facial profiles, lip incompetence, dental crowding and marked lip inclination, causing changes in facial esthetics, mastication, and speech problems (8). Facial and occlusal harmony is associated with several etiological factors such as persistent digit-sucking habits, posture, respiratory problems, tongue size, and condylar problems that are often present in AOB (9).

Some publications have pointed out that the quality of life of patients with AOB has been affected by the oral condition (10,11), and that this is also related to sex and age, affecting them psychologically and socially in their environment (12). An important aspect to consider is the age group; for example, in adolescents, concern for their physical appearance increases and they are more interested in social acceptance, significantly affecting their self-esteem and social relationships (13-14). At this stage, if the adolescent is exposed to AOB, it could affect his or her quality of life in terms of oral health during swallowing, pronunciation, smiling and social development due to the esthetic impact it has (15).

The prevalence of malocclusions in school children varies widely, some consider a range between 4.93%, 7.1%, into 9%, so the impact that these can have on their quality of life is something that should be considered (16-18). Currently there is some research that relates the impact on quality of life in patients with anterior open bite. Others conclude that malocclusions significantly affect interpersonal relationships and self-esteem (19,20). However, to date there is no systematic review that analyzes the influence of open bite on quality of life. Therefore, the purpose of the present systematic review was to determine the influence of AOB on oral health-related quality of life.

## Material and Methods

-Protocol and registration

The present systematic review was registered in the research and ethics commission of the Universidad Cientifica del Sur with registration code No. PRE-8-2022-00603 and was also registered in the INPLASY database with registration code 202370071 (https://inplasy.com/payment-inplasy-ct/). The review followed the structure according to the preferred reporting elements guidelines for systematic reviews and meta-analysis (PRISMA). 

-Eligibility criteria

The research question was formulated using the PECOs strategy for the selection of the studies. The selection criteria were.

1. Participants: Children, adolescents, and adults with anterior open bite.

2. Exposition: Diagnostic of anterior open bite malocclusion.

3. Comparison: Diagnosis of adequate bite.

4. Outcomes: Quality of life related to oral health.

5. Types of Study: Cross-sectional, case-control and cohort studies.

-Search strategies

The search strategies were applied in the following electronic databases until September 23, 2023: MEDLINE (via PubMed), EBSCO, SCOPUS, and LILACS. The search strategies were limited to individuals, without restriction of publication time, in Spanish, English or Portuguese. Search terms (strategies) were developed for MEDLINE (via PubMed), SCOPUS, EBSCO and LILACS as shown in  Table 1.

-Study selection 

The selection of studies was performed in two periods. In the first period two authors (JRH and GVM) analyzed the titles and/or abstracts indistinctly, also including references that met the eligibility criteria focusing on children, adolescents, and adults due to the different stage transitions. Papers of low relevance with little information and that did not focus on those age groups were excluded. In the second phase, the authors themselves independently reviewed the full texts, including articles that met the selection standards; in case of discrepancy by the authors, the inclusion of the article was determined with the help of a third investigator (LEAG).

-Quality assessment

A modified version of the Newcastle Ottawa tool (21) was used to assess the risk of bias of the selected studies. The following items were assessed: representativeness of the sample, nonrespondents, exposure ascertainment, comparability, outcome, statistical test. Bias was considered in each item and the overall risk of bias of the studies was assessed as high, moderate, and minimal risk information. https://www.ohri.ca/programs/clinical_epidemiology/oxford.asp 

## Results

**Figure 1:**
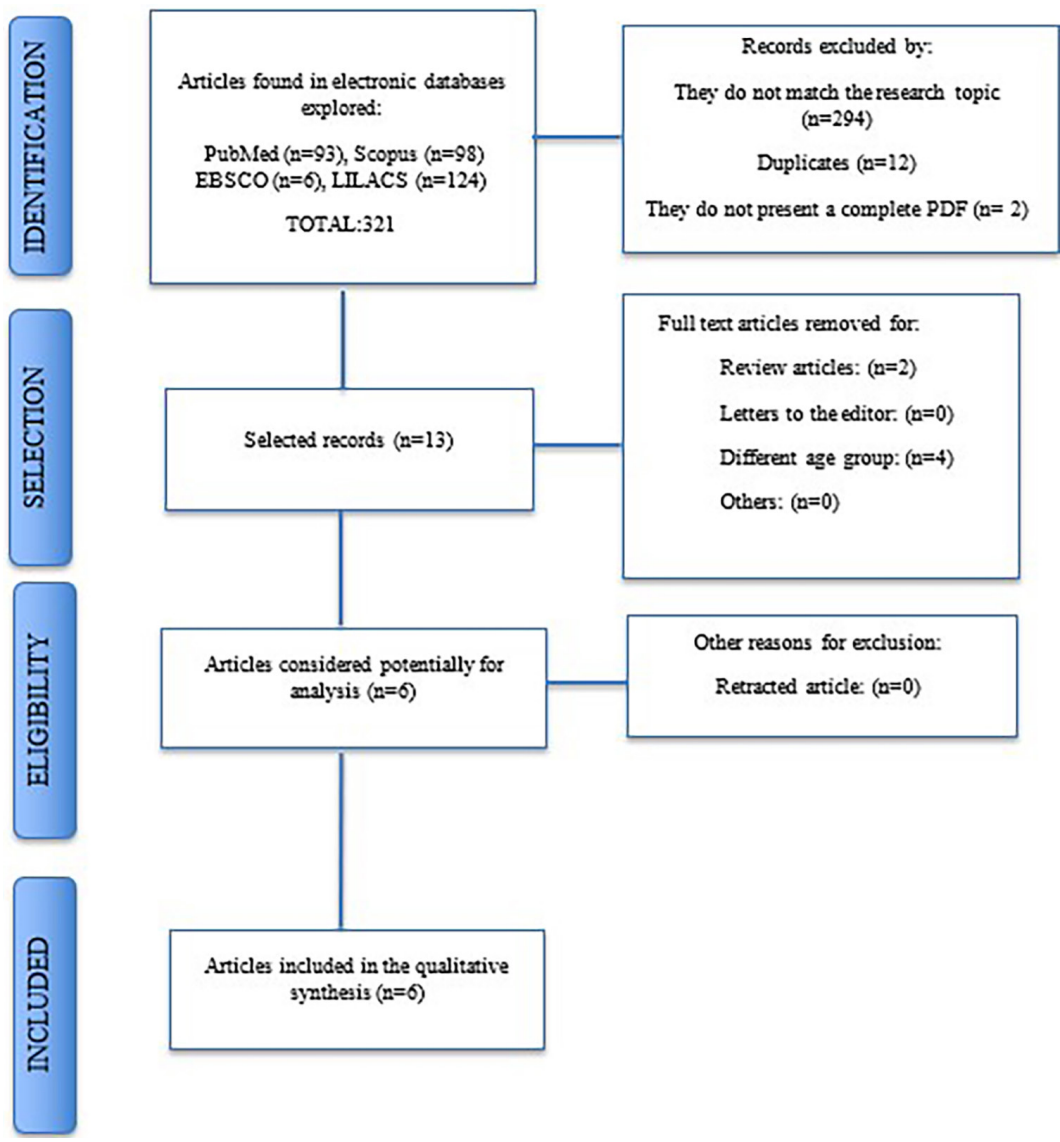
Flowchart of the selected studies.

The search strategy found a total of 321 articles in the databases. After applying the criteria for inclusion and elimination of duplicate articles, six articles were selected for full-text evaluation. A search flow diagram explaining this selection is provided in Figure 1.

-General characteristics of the articles analyzed

 Table 2 describes the general characteristics of the studies. The six studies selected were cross-sectional studies. Regarding population, the study by Masood *et al*. (22) (n=4711) obtained a larger population compared to the other 5 studies Curto *et al*. (23) (n=80), Da Rosa *et al*. (24) (n=478), Gomez *et al*. (25) (n=769), Swan *et al*. (26) (n=240), Ramos *et al*. (27) (n=504). All studies collected survey data to find the relationship between anterior open bite and patients’ quality of life.

-Risk of bias

**Figure 2:**
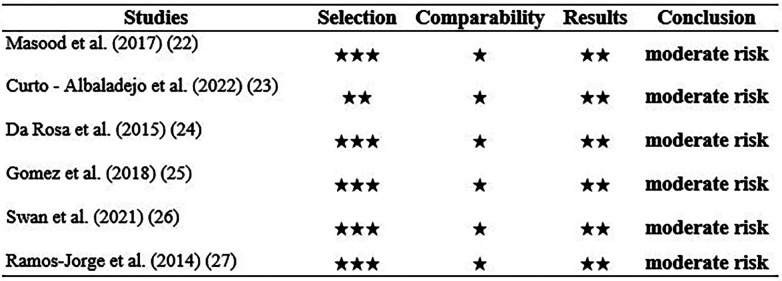
Risk of Bias of the selected studies.

Regarding the risk of bias, the six studies were considered to have a moderate risk of bias, (Fig. 2). Regarding the representativeness of the sample in the studies by Masood *et al*. (22), da Rosa *et al*. (24), Gomez *et al*. (25), Swan *et al*. (26), Ramos *et al*. (27), there was random sampling, and we consider that the sample size in general is adequate, except for the study by Curto *et al*. (23), which presents a study selection by convenience. Likewise, the six studies do not present information on the people who were surveyed. All studies used a validated measurement tool, and conducted a self-report provided by each patient on the impact of AOB on quality of life. An important detail is that the six studies clearly detailed the results analyzed using the corresponding statistical tests.

-Results of the studies analyzed

 Table 3 describes the influence of AOB on quality of life, in the six studies the evaluation parameters were similar such as functional limitation, physical pain, psychological discomfort, physical disability, psychological disability, social disability. In the study of Masood *et al*. (22) and Curto *et al*. (23) the evaluation is performed in adults. In the study by Curto *et al*. (23) in both groups evaluated there is no significant difference, however, it is evident that anterior open bite has more influence on the quality of life of women: (functional limitation: 1.21, physical pain: 1.18, psychological discomfort: 1.15, physical disability: 0.32, psychological disability: 1.55, social disability: 0.20) than of men: (functional limitation: 1.21, physical pain: 1.16, psychological pain: 1.16, social disability: 0.20). 21, physical pain: 1.16, psychological discomfort: 1.24, physical disability: 0.28, psychological disability: 1.59, social disability: 0.10), while the study by Masood *et al*. (22) shows that there is also an impact on the quality of life (functional limitation: 0.90, physical pain: 1.0, psychological level: 1.02, physical discomfort: 0.7, psychological disability: 1.26, social disability: 0.79) of people. In the study of Da Rosa *et al*. (24), Gomez *et al*. (25), Swan *et al*. (26) and Ramos *et al*. (27) evaluated the influence of AOB on quality of life in children. In the study by Da Rosa *et al*. (24) it was found that the AOB negatively affected the quality of life in symptomatology: children without treatment: 1.58 and with treatment: 1.70, and in function: children without treatment: 2.37 and children with treatment: 2.24, also in the study by Gomez *et al*. (25) there is evidence of a negative impact of anterior open bite on the quality of life at the psychological level in children without treatment: 2.00 and in children with treatment: 1.62. In the study by Ramos *et al*. (27) anterior open bite has a negative impact on the quality of life of children in the parameters of symptomatology, function, psychological level, and social interaction with other people, however, there is a greater impact on: function: 3.77 and psychological level: 2.26. In the study of Swan *et al*. (26), *et al*. showed in comparison with the other studies that there is no influence of anterior open bite on the quality of life showing that there is no significant difference between boys: (functional limitation: 0.11, emotional wellbeing: -0.06, social wellbeing: -0.02) and girls: (functional limitation: -0.02, emotional wellbeing: -0.10, social wellbeing: -0.04).

## Discussion

The quality of life-related to oral health is a crucial topic in orthodontics. Orthodontic treatments aim to restore optimal dental occlusion and enhance quality of life in areas such as smiling, swallowing, and speech. Certain malocclusions, particularly AOB, can significantly impact these aspects. AOB affects the tongue’s position against the anterior part of the hard palate, leading to negative consequences for dental function and aesthetics. Therefore, this systematic review aims to assess the influence of AOB on oral health-related quality of life. The objective is to understand how this malocclusion impacts patients’ lives and which dimensions are most affected.

The results of this systematic review are derived from six studies. Even though a thorough search was conducted using primary information sources, the established selection criteria limited the number of studies included. Although there was a limitation, the identified bias was primarily moderate, which hindered reaching definitive conclusions. However, these results provide a general understanding of the relationship between the variables and suggest hypotheses for further investigation. Dentists should consider these insights in their daily practice.

Regarding the influence of AOB on quality of life, there is a pattern that is repeated in most of the studies evaluated, which are the psychological factor and the functional factor; that is, it directly affects patients either in mental or physical well-being and, in, turn affects the activities they perform daily during speech, eating and smiling. In this sense, in the studies of Masood *et al*. (22) and Curto *et al*. (23), who evaluated adults, they found the influence of this malocclusion on their quality of life. They argued that this may be because they have multiple responsibilities and pressures they face in different areas of their lives, which makes them particularly vulnerable to the influence of psychological factors affecting both their mental health and their daily performance.

Likewise, the studies by Da Rosa *et al*. (24), Gomez *et al*. (25) and Ramos *et al*. (27) identify that there is a significant impact on the quality of life of the children evaluated, these authors explain that these results are due to their vulnerability in their developmental stages. They highlight this critical stage of their life in which they are learning to manage their emotions and develop cognitive and social skills, unlike adults, children do not yet have the psychological tools or skills to manage stressful situations and they form their perceptions of themselves based on interactions with their environment including opinions of their parents, teachers, or peers. Thus, if a child experiences rejection, bullying, or constant criticism of their physical appearance, this can lead to self-esteem problems or depression, affecting their emotional functioning. However, the study by Swan *et al*. (26) showed that there is no impact of AOB on the quality of life of adolescents; this may be because the sociocultural and psychological level of the country of the patients in that study is different from the rest of the countries, which may affect their quality of life to a lesser extent, this agrees with the findings of Bonomi *et al*. (28) who mentioned that the quality of life-related to oral health depends mostly on cultural influences so that patients have different perceptions and interpretations on this issue.

It is important to note that the studies analyzed various parameters to assess how AOB affects quality of life. These parameters included psychological and functional aspects, physical pain, physical disability, and social interaction. This comprehensive approach allows for a more precise measurement of the influence of AOB on quality of life. Thus, there was a common denominator in the conclusions found in five of the six studies analyzed, indicating an influence of the AOB on the quality of life of the affected persons in the mentioned components. Even though the methodology of the analyzed studies presents a moderate level of bias, the uniformity of the answers and their methodological strategies allow us to manage a trend in the results despite having to be evaluated in better-designed studies.

One limitation of this systematic review is that a meta-analysis could not be conducted, as the selected studies measured quality of life differently. Consequently, this statistical tool was not applicable. However, the similarity of the results enables us to assess a typical response across the evaluated studies.

## Conclusions

The studies selected for this systematic review were mainly of moderate bias quality. A majority consensus found that AOB influences oral health-related quality of life, mainly in the psychological and functional factors; in other words, it mainly affects the daily dimensions of speech, eating, and smiling. This situation should be considered necessary by orthodontists and patients presenting this condition, even though more and better-designed studies should be carried out.

## Figures and Tables

**Table 1:** Search strategy in different databases.

Electronic data bases	(Key Words)
PubMed	"quality of life"[All Fields] AND "Oral Health"[All Fields] AND ("Open Bite"[All Fields] OR "Nonocclusion"[All Fields] OR "anterior open bite"[All Fields]) ALL ( "Quality of Life" AND "Oral Health" AND "Open Bite" ), "quality of life" AND "Oral Health" AND "Open Bite" ("Quality of Life" OR "Life Quality" OR "Health-Related Quality Of Life" OR "Health Related Quality Of Life" OR "HRQOL"), ("open bite" OR "Bite, Open" OR "Nonocclusion" OR "Openbite" OR "Apertognathia")
Scopus	"quality of life"[All Fields] AND "Oral Health"[All Fields] AND ("Open Bite"[All Fields] OR "Nonocclusion"[All Fields] OR "anterior open bite"[All Fields]) ALL ( "Quality of Life" AND "Oral Health" AND "Open Bite" ), "quality of life" AND "Oral Health" AND "Open Bite" ("Quality of Life" OR "Life Quality" OR "Health-Related Quality Of Life" OR "Health Related Quality Of Life" OR "HRQOL"), ("open bite" OR "Bite, Open" OR "Nonocclusion" OR "Openbite" OR "Apertognathia")
Ebsco	"quality of life"[All Fields] AND "Oral Health"[All Fields] AND ("Open Bite"[All Fields] OR "Nonocclusion"[All Fields] OR "anterior open bite"[All Fields]) ALL ( "Quality of Life" AND "Oral Health" AND "Open Bite" ), "quality of life" AND "Oral Health" AND "Open Bite" ("Quality of Life" OR "Life Quality" OR "Health-Related Quality Of Life" OR "Health Related Quality Of Life" OR "HRQOL"), ("open bite" OR "Bite, Open" OR "Nonocclusion" OR "Openbite" OR "Apertognathia")
LILACS	"quality of life"[All Fields] AND "Oral Health"[All Fields] AND ("Open Bite"[All Fields] OR "Nonocclusion"[All Fields] OR "anterior open bite"[All Fields]) ALL ( "Quality of Life" AND "Oral Health" AND "Open Bite" ), "quality of life" AND "Oral Health" AND "Open Bite" ("Quality of Life" OR "Life Quality" OR "Health-Related Quality Of Life" OR "Health Related Quality Of Life" OR "HRQOL"), ("open bite" OR "Bite, Open" OR "Nonocclusion" OR "Openbite" OR "Apertognathia")

**Table 2:** General characteristics of the analyzed articles (n = 6).

Author and year	Study design and type	Population	Eligibility criteria	Variables measured	QOL measurement instrument	Evaluated dimensions of QOL	Measuring AOB
Masood et al. (2017) (22)	Observational, analytical (cross-sectional)	A total of 4711 people was included	Inclusion: -adults aged ≥30 years.	-Quality of life -Open bite	Oral Health Impact Profile-14 (OHIP-14)	D1: Functional limitation D2: Physical pain D3: Psychological discomfort D4: Physical disability D5: Psychological D6: Social disability	Presence and absence of anterior open bite in adults, also analyzing the influence of sex and age on quality of life
Curto et al. (2022) (23)	Observational, transversal	80 adults (men: 34 and women: 46) (40 case) (40 control)	Inclusion: - + 18 years - no craniofacial abnormalities - no missing teeth - no previous orthodontics and/or dentofacial orthopedic treatment. Exclusion: - Patients with untreated caries - Patients with gingival and/or periodontal pathologies - Patients with severe dentofacial abnormalities.	-Quality of life -Open bite	Oral Health Impact Profile-14 (OHIP-14)	D1: Functional limitation D2: Physical pain D3: Psychological discomfort D4: Physical disability D5: Psychological disability D6: Social disability D7: Disadvantage	Presence and absence of anterior open bite in adults, also analyzing the influence of sex and age on quality of life
Da Rosa, et al. (2015) (24)	Observational, transversal	478 children from 12 to 59 months	Malocclusion in the anterior region: anterior open bite, overjet and labial seal.	-Quality of life -Open bite	Early Childhood Oral Health Impact Scale (ECOHIS)	D1: symptoms D2: function D3: psychological, self-image/social interaction domains	The impact of anterior open bite on the quality of life of preschool children
Gomez et al. (2018) (25)	Observational, analytical (cross-sectional)	Total 764 pairs of children and parents	Inclusion: -five-year-old children -no systemic diseases -enrolled in public and private preschools. Exclusion: -the presence of one or more erupted permanent teeth and a history of orthodontic treatment.	-Quality of life -Open bite	-The Scale of Oral Health Outcomes for Five-Year-Old Children (SOHO-5) -Sense of coherence (SOC-13)	D1: Difficulty eating D2: Difficulty speaking D3: Difficulty playing D4: Difficulty sleeping D5: Avoiding smiling due to pain D6: Difficulty smiling due to appearance	To evaluate the association between psychological factors (SOC, LOC and OHRQoL), sociodemographic conditions, oral habits and anterior open bite
Swan et al. (2021) (26)	Observational, analytical (cross-sectional)	Total, of 240 participants, boys 114 (47.5%) girls 126 (52.5%)	Inclusion: -adolescents seeking orthodontic treatment -any race and ethnicity who were treated with fixed appliances had to be between 11 and 14 years old Exclusion: -patients with congenital anomalies or craniofacial deformities such as cleft lip and palate	-Quality of life -Open bite	-The Orthodontic Quality of Life Assessment Survey (OQoLAS)	D1: Functional limitation D2: Physical pain D3: Psychological discomfort D4: Physical disability D5: Psychological D6: Social disability	the correlation between the orthodontic quality of life of adolescents and the objective complexity of the case measured by the ABO-DI analyzed by sex and age.
Ramos et al. (2014) (27)	Observational, analytical (cross-sectional)	499 (start), 451 (end) 53.9% women	Inclusion: -Primary school students -Without distinction based on sex -Ages between 3-5 years Exclusion: -Being under orthodontic treatment and systemic disease	-Quality of life -Open bite	Early Childhood Oral Health Impact Scale (ECOHIS)	Symptoms domain (SD) Function domain (FD)	Presence and absence, Previous open bite was recorded in the absence of a vertical overlap of the incisors in the occlusal position.

**Table 3:** Results of the studies analyzed.

Author and year	Impact on quality of life					Conclusions
		Male	Female	P	Total	
Masood et al. (2017) (22)		Adults in general:				Anterior open bite had a greater impact on psychological disability in adults and affected quality of life.
	• Functional limitation	0.9				
	• Physical pain	1.0				
	• Psychological	1.02				
	• Physical discomfort	0.07				
	• Psychological disability	1.26				
	• Social disability	0.79				
Curto et al. (2022) (23)	• Functional limitation	1.21	1.21	0.806	0	There was no significant difference between the two groups, however, due to the scores recorded, it is evident that in women there is more influence of the anterior open bite on their quality of life.
	• Physical pain	1.16	1.18	0.642	0.002	
	• Psychological discomfort	1.24	1.15	0.309	0,018	
	• Physical disability	0.28	0.32	0.726	0.002	
	• Psychological disability	1.59	1.55	0.69	0.001	
	• Social disability	0.1	0.2	0.318	0.025	
Da Rosa et al. (2015) (24)		Untreated children:	Children with treatment:			It was shown that AOB negatively affects the quality of life in the symptoms and stomatological function of children without treatment and with treatment.
		1.58	1.7			
	• Symptoms	2.37	2.24			
	• Function					
Gomez et al. (2018) (25)		Untreated children:	Children with treatment:			It was shown that AOB negatively affected the psychological factor of children in their quality of life and there was a greater impact on children who did not receive treatment compared to children who did receive treatment.
	• Psychological factor	2	1.62			
		
Swan et al. (2021) (26)	• Functional limitation	children: 0,11	girls: -0,02	0.9197	Boys: -0.00	There is no significant difference, therefore there is no influence of anterior open bite on the quality of life of adolescents.
	• Emotional well-being	children: -0,06	girls: -0,10	0.9424	Girls: -0.07	
	• Social well-being	children: -0,02	girls: -0,04	0.9961		
Ramos-Jorge et al. (2014) (27)		Children in general:			2.55	AOB negatively affects the quality of life of children, showing a greater impact on function and psychological level.
	• Symptoms	1.12				
	• Function	3.77				
	• Psychological	2.26				
	• Social interaction	1.67				

## Data Availability

The datasets used and/or analyzed during the current study are available from the corresponding author.
